# Supplementary material for: Development of Mode-Switchable Touch Sensor Using MWCNT Composite Conductive Nonwoven Fabric
Source: Polymers (Basel). 2022 Apr 11;14(8):1545. doi: 10.3390/polym14081545 (PMC9031523; doi:10.3390/polym14081545)
Supplement: Supplementary file 1 [file polymers-14-01545-s001.zip › polymers-1609309 supplementary material.pdf]

polymers

Supporting Information

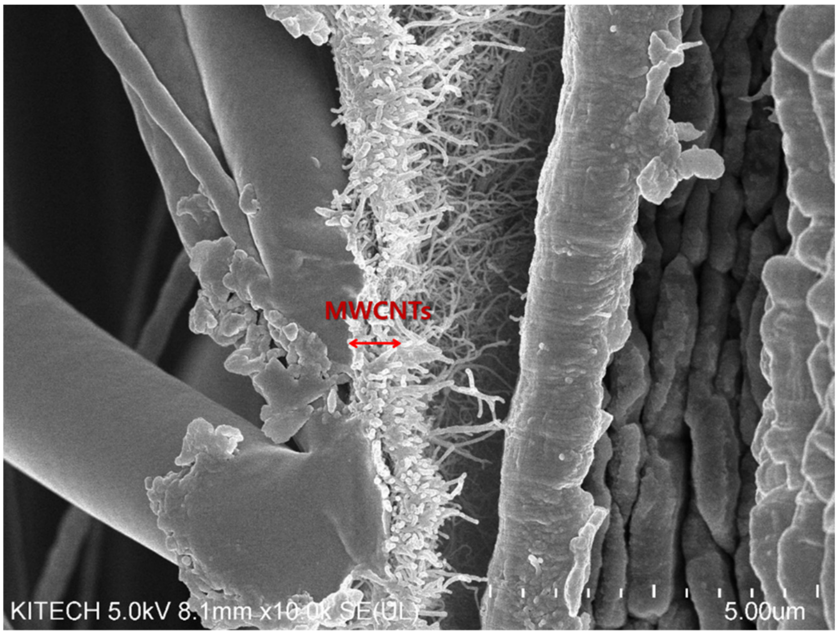

Supplementary Figure S1. SEM cross-section image of the prepared conductive nonwoven for analyzing the thickness of the coated MWCNTs.

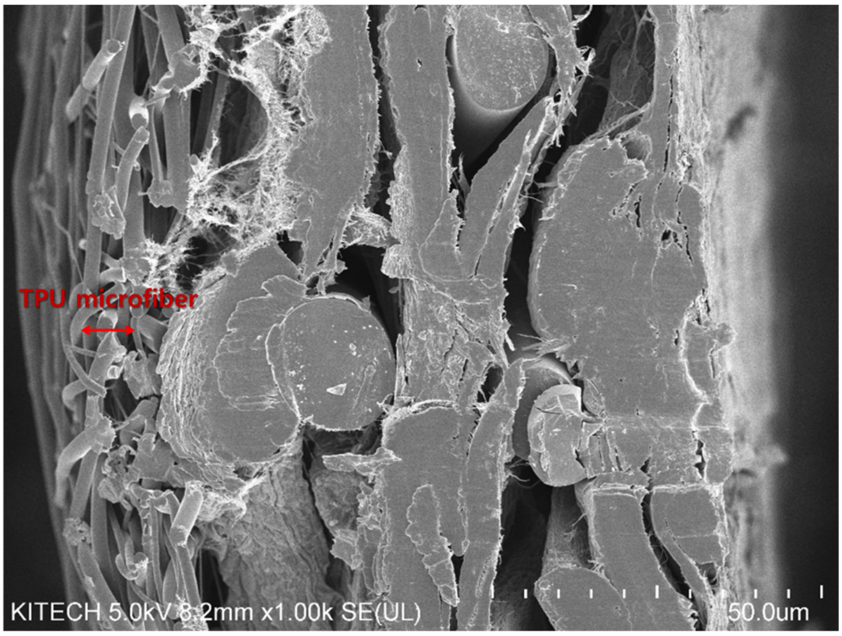

Supplementary Figure S2. SEM cross-section image of the prepared conductive nonwoven for analyzing the thickness of the electrospun TPU microfibers.

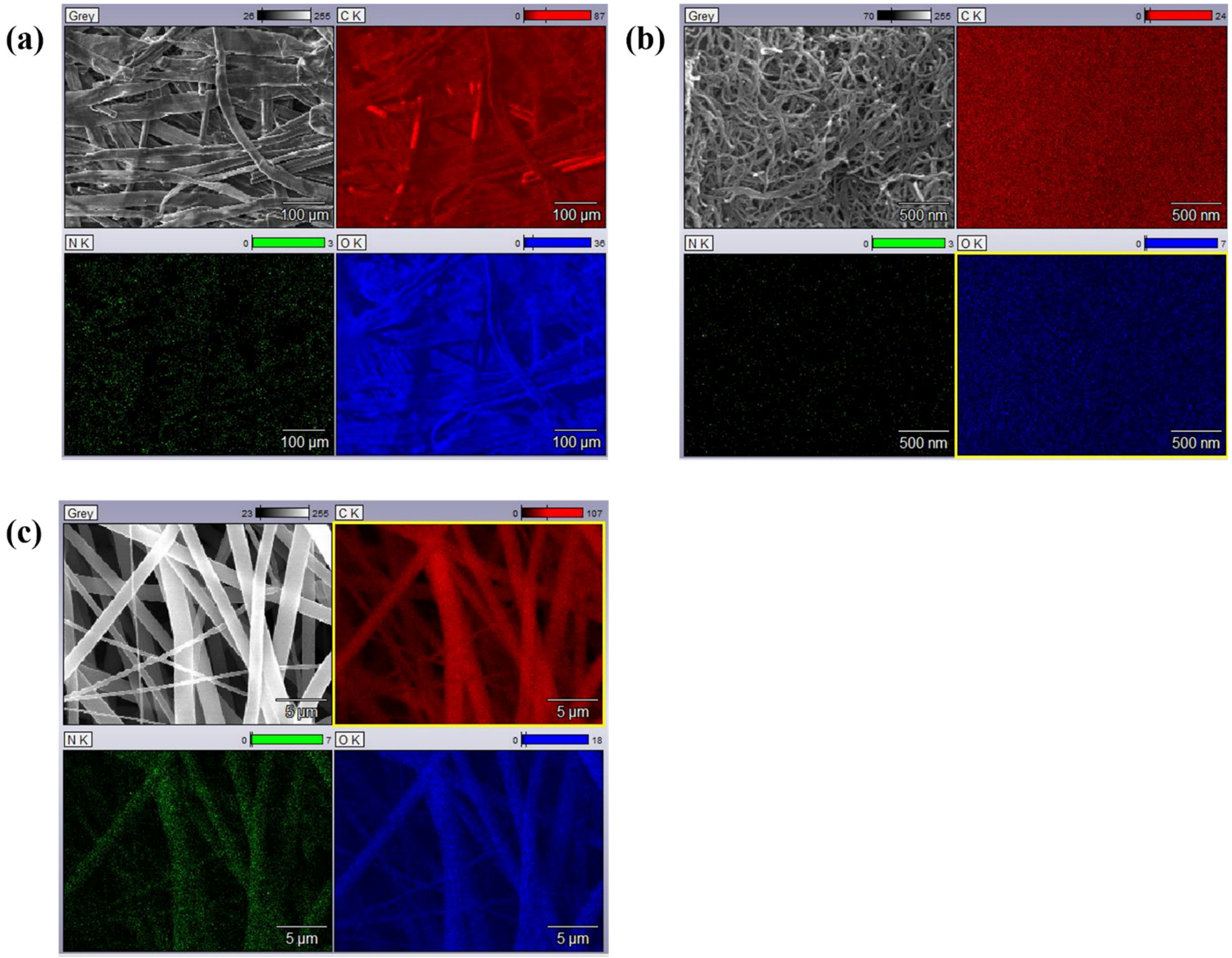

Supplementary Figure S3. Energy-dispersive X-ray spectroscopy (EDS) mapping of MWCNT composite conductive nonwoven: (**a**) CNwCa SEM image, including C element, N element, and O element; (**b**) MWCNTs on CNwCa SEM image, including C element, N element, and O element; (**c**) TPU microfibers on MWCNTs + CNwCa SEM image, including C element, N element, and O element.

Supplementary Table S1. Resistance with respect to the MWCNT content after the spraying process.

MWCNT content	Add-on wt%		Add-on wt%		Add-on wt%		Add-on wt%	
	1.3		2.6		5.2		7.8	
	without CNwCa	on CNwCa	without CNwCa	on CNwCa	without CNwCa	on CNwCa	without CNwCa	on CNwCa
Re-sistance (Ω)	814	592	811	330	475	292	201	110
	948	486	918	323	495	235	210	118
	965	509	975	368	499	250	211	120
	1010	522	1030	369	502	268	217	129
	1080	599	1050	388	581	263	244	149
	990	615	957	330	482	263	202	110
	1035	609	826	358	535	265	230	121
	1054	610	925	387	596	280	252	122
	850	620	856	395	599	277	253	127
	950	574	884	376	562	289	243	140
Average	970	574	923	362	533	268	226	125
Standard deviation	80.6	46.8	77.1	25.0	45.8	16.5	19.5	11.7
CV%	8.3	8.2	8.4	6.9	8.6	6.1	8.6	9.4

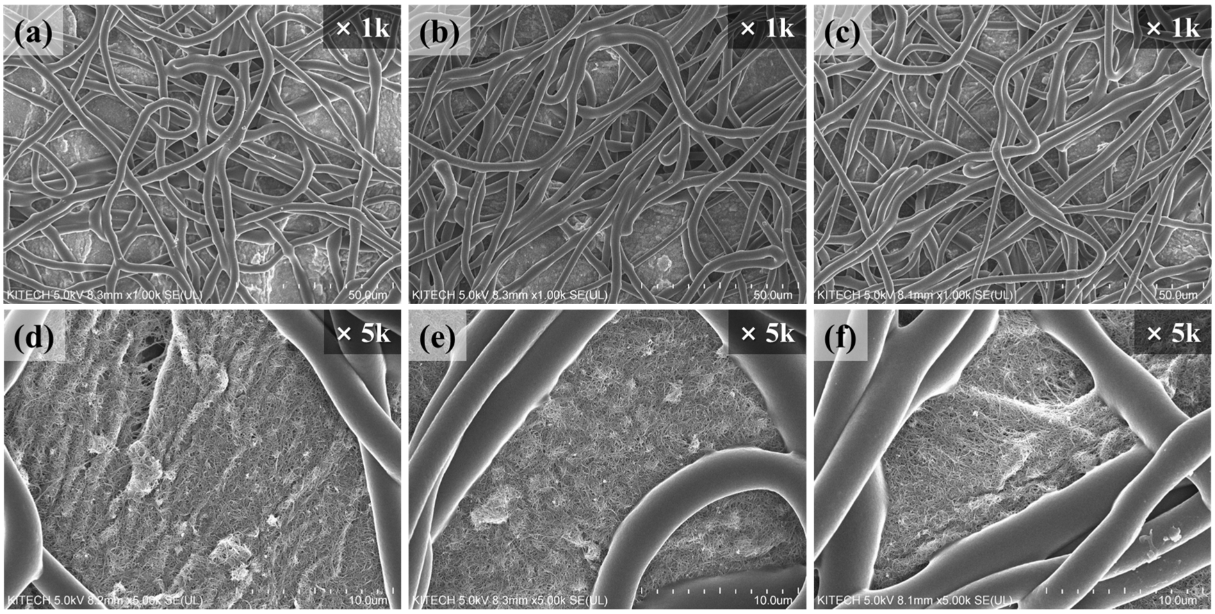

Supplementary Figure S4. SEM images of the conductive nonwoven surface at various electrospinning durations: (**a**) 10 min, (**b**) 15 min, (**c**) 20 min; (**d–f**) partially enlarged images of (**a–c**), respectively.

Supplementary Table S2. Resistance with respect to the electrospinning duration of the conductive nonwoven surface.

Electrospinning duration	Ref.	10 min.	15 min.	20 min.
Resistance (Ω)	292	566	871	1974
	235	599	883	1978
	250	603	937	1444
	268	611	1061	1354
	263	633	1002	1567
	263	660	978	1656
	265	628	983	2204
	280	565	1067	2078
	277	701	970	1593
	289	608	1105	1750
Average	268	617	986	1761
Standard deviation	16.5	39.0	72.9	270.6
CV%	6.1	6.3	7.4	15.4

Supplementary Table S3. Resistance value of the conductive nonwoven before and after washing.

No.	1	2	3	4	5	6	7	8	9	10	11	12	13	14	15	16	17	18
Before washing	566	599	603	611	630	660	628	565	701	608	571	685	668	704	656	562	581	571
After washing	653	689	620	642	642	688	701	591	573	558	579	703	664	620	584	691	653	664
No.	19	20	21	22	23	24	25	26	27	28	29	30	31	32	33	34	35	36
Before washing	601	562	601	645	581	591	702	633	633	622	601	600	668	645	656	633	602	562
After washing	676	599	610	653	642	611	580	589	653	593	593	561	673	688	631	622	631	694
No.	37	38	39	40	41	42	43	44	45	46	47	48	49	50	**Av.**	**STEV.**		**CV %**
Before washing	571	612	581	633	601	612	592	601	622	633	668	591	602	633	**617**	**37.9**		**6.1**
After washing	688	676	620	599	589	552	620	552	580	603	600	688	599	570	**627**	**44.3**		**7.1**

Supplementary Table S4. Resistance value of the MWCNT composite conductive nonwoven before and after folding.

Number of times folded	0	1000	2000	3000	4000	5000
Resistance(Ω)	566	555	572	572	543	526
	599	601	601	615	614	705
	603	603	611	603	627	619
	611	611	620	621	625	687
	633	645	642	643	646	649
	660	673	666	670	683	696
	628	630	634	638	643	644
	565	567	541	565	549	596
	701	703	707	711	735	757
	608	600	599	598	694	578
Average	617	619	619	624	636	646
Standard deviation	39.0	43.1	44.5	42.0	56.9	65.0
CV%	6.3	7.0	7.2	6.7	9.0	10.1

Supplementary Table S5. Datasheet of the load–resistance calibration curve.

Load (gf/cm²)	4.0	7.7	13.5	15.0	22.3	29.6	36.9	51.5	73.4	88.0
Resistance(Ω)	1059	832	566	599	429	388	340	227	157	99
	1050	706	599	493	432	312	304	220	155	110
	1159	698	603	505	436	334	296	224	154	143
	1200	785	611	572	444	381	343	232	162	144
	970	742	633	529	466	348	300	247	177	109
	1148	847	660	580	493	379	351	251	181	129
	1158	725	628	512	461	341	283	249	179	138
	1160	798	565	585	408	374	336	223	153	115
	1060	796	701	583	504	372	344	292	212	123
	944	704	608	546	437	358	327	228	158	139
Average	1091	763	617	550	451	359	322	239	169	125
Standard deviation	83.0	52.4	39.0	36.4	28.4	23.2	23.1	20.7	17.7	15.3
CV%	7.6	6.9	6.3	6.6	6.3	6.5	7.2	8.6	10.5	12.2
